# Chitin protects the gut epithelial barrier in a protochordate model of DSS-induced colitis

**DOI:** 10.1242/bio.029355

**Published:** 2017-12-08

**Authors:** Assunta Liberti, Ivana Zucchetti, Daniela Melillo, Diana Skapura, Yoshimi Shibata, Rosaria De Santis, Maria Rosaria Pinto, Gary W. Litman, Larry J. Dishaw

**Affiliations:** 1 Department of Animal Physiology and Evolution, Stazione Zoologica Anton Dohrn, Napoli 80121, Italy; 2 University of South Florida, Morsani College of Medicine, Department of Pediatrics, Tampa, FL 33606, USA; 3 Institute of Protein Biochemistry (IBP), National Research Council (CNR), Napoli 80131, Italy; 4 Molecular Genetics, Johns Hopkins All Children's Hospital, Saint Petersburg, FL 33701, USA; 5 Biomedical Science Department, Florida Atlantic University, Charles E. Schmidt College of Medicine, Boca Raton, FL 33431, USA

**Keywords:** Inflammatory bowel disease, DSS-induced colitis, *Ciona intestinalis*, Chitin-rich mucus, VCBPs, Host-microbes interaction

## Abstract

The gastrointestinal tract of *Ciona intestinalis*, a solitary tunicate that siphon-filters water, shares similarities with its mammalian counterpart. The *Ciona* gut exhibits other features that are unique to protochordates, including certain immune molecules, and other characteristics, e.g. chitin-rich mucus, which appears to be more widespread than considered previously. Exposure of *Ciona* to dextran sulphate sodium (DSS) induces a colitis-like phenotype similar to that seen in other systems, and is characterized by alteration of epithelial morphology and infiltration of blood cells into lamina propria-like regions. DSS treatment also influences the production and localization of a secreted immune molecule shown previously to co-localize to chitin-rich mucus in the gut. Resistance to DSS is enhanced by exposure to exogenous chitin microparticles, suggesting that endogenous chitin is critical to barrier integrity. Protochordates, such as *Ciona*, retain basic characteristics found in other more advanced chordates and can inform us of uniquely conserved signals shaping host-microbiota interactions in the absence of adaptive immunity. These simpler model systems may also reveal factors and processes that modulate recovery from colitis, the role gut microbiota play in the onset of the disease, and the rules that help govern the reestablishment and maintenance of gut homeostasis.

## Abstract (precis)

**Summary:** The protochordate, *Ciona intestinalis*, is defining conserved properties of gut mucosal barriers and inflammatory processes. Diverse model systems can benefit studies concerning human pathologies of the gut.

## INTRODUCTION

The gastrointestinal tract (GIT) represents an external environment that runs through the otherwise sterile confines of an animal. By ingesting food and water, animals expose the GIT to countless antigens and microorganisms, many of which can be metabolized as part of the diet whereas others become permanent occupants. Gut-dwelling microorganisms that become part of the host microbiota can either remain neutral or serve beneficial or detrimental roles to host health. Owing to the continuous exposure to outer stimuli, the gut has evolved strategies to avoid pathogenic infections and maintain homeostasis, resulting in a balance (i.e. homeostasis) between host immunity and gut microbiota (Hooper and Macpherson, 2010). Dysbiosis, the loss of this balance, is characterized by an inappropriate and aberrant immune response to microbes that is the underlying cause of inflammatory bowel disease (IBD) pathologies, such as ulcerative colitis and Crohn's disease (Matsuoka and Kanai, 2015; Swidsinski et al., 2002).

The experimental induction of colitis has been used as a way to study not just gut inflammatory processes that often contribute to autoimmune phenomena, but also to understand the processes that shape barrier integrity and homeostasis. To induce colitis, a variety of approaches have been developed, including the administration of chemicals such as acetic acid, formalin, indomethacin, trinitrobenzene sulfonic acid, oxazolone and nonsteroidal anti-inflammatory drugs. Polysaccharides, such as dextran sulphate sodium (DSS), that physically and/or functionally disrupt gut barriers also have been employed for this purpose (Gaudio et al., 1999; Randhawa et al., 2014). DSS is by far the most commonly used and has been shown to induce colitis in diverse animal models producing phenotypes and associated inflammatory profiles that resemble human IBDs. The addition of DSS to drinking water generates colitis in the mouse (Chassaing et al., 2014; Okayasu et al., 1990; Perše and Cerar, 2012; Rose et al., 2012a; Wirtz et al., 2007, 2017), rat (Gaudio et al., 1999), pig (Kim et al., 2009), and the fruit fly, *Drosophila melanogaster* (Amcheslavsky et al., 2009). The addition of DSS to water for studies involving exposure by immersion has been developed in zebrafish larvae (Oehlers et al., 2012, 2013); immersion was found to also result in the induction of an IBD-like colitis. The application of diverse model systems for experimentally - induced colitis offers specific advantages for understanding the phenotypes and physiology associated with IBD-like pathologies.

*Ciona intestinalis*, a well-recognized developmental model system, has been shown to be uniquely informative for studies of host-microbe interactions in the gut (Dishaw et al., 2012, 2016). *Ciona* lacks adaptive immunity (Azumi et al., 2003), relying solely on innate immunity for host defense, and maintains a core microbiome (Dishaw et al., 2014b) that likely is influenced through immune interactions involving chitin-rich mucus that coats the gut epithelium (Dishaw et al., 2016). It was previously hypothesized that the interwoven chitin fibers, produced endogenously and incorporated into gut mucus, could enhance barrier functions in the *Ciona* gut (Dishaw et al., 2016). Thus, *Ciona* represents a potentially informative model system to define how barrier integrity shapes the maintenance of homeostasis between host and microbiome.

In order to address the roles that endogenous chitin-rich mucus may be serving in barrier defenses, we have developed approaches to implement DSS treatment via immersion as a means to challenge mucosal barriers and for studying associated inflammatory responses. We find that DSS-treatment in *Ciona* induces a colitis-like phenotype that is remarkably similar to that seen in mammals. Pre-treatment with exogenous chitin microparticles (CMPs) can protect the animal from this DSS-mediated damage. While chitin-rich mucus is not normally found in mammals, it was shown previously that administration of CMPs to mice, prior to DSS treatment, could afford protection from inflammatory colitis (Nagatani et al., 2012). Collectively these findings suggest that endogenous chitin likely serves a role in enhancing barriers, and underscore the utility of the *Ciona* model system in the identification of evolutionarily conserved features as well as taxa-specific innovation that shape mucosal barriers and associated innate immune responses.

## RESULTS

### DSS treatments induce gut epithelium damage in *Ciona* adults and juveniles

Preliminary experiments were undertaken to define the most appropriate sub-lethal DSS concentration affecting the digestive tract morphology and physiology, as observed in other animal models. It has been established that DSS concentration ranging between 1% and 0.5% does not impair animal viability (Fig. S1). Indeed, immediately after immersion of the animals in DSS-containing filtered seawater (FSW), the first noticeable change compared to control animals was the vacating of the gut with the release of loose stools into the water.

**Fig. 1.**
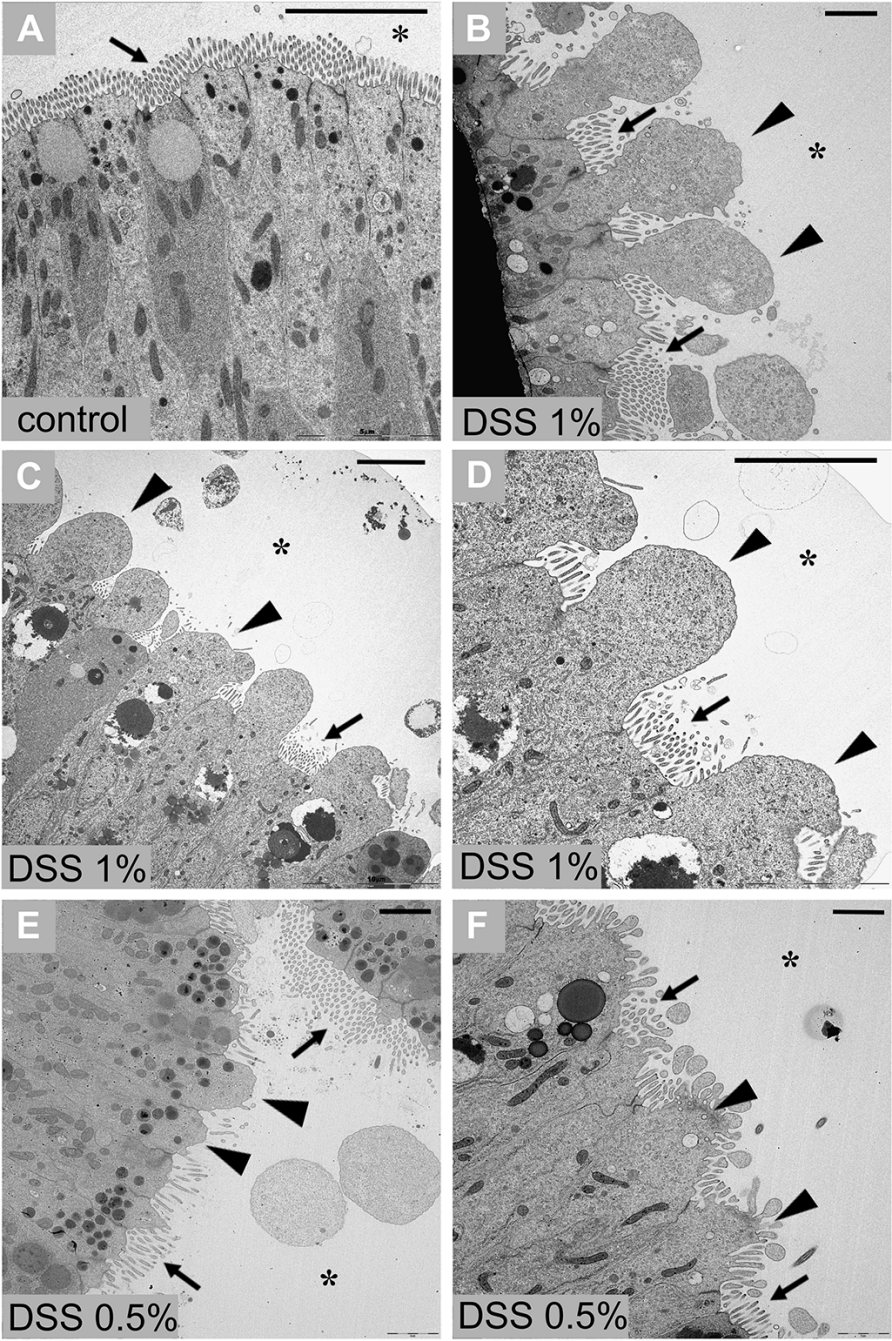
**TEM of stomach epithelium of adult *Ciona*.** Stomach epithelium of control animal, not treated with DSS (A). Stomach epithelium of animals treated with 1% DSS (B-D) and 0.5% DSS (E-F) exhibits morphological alterations that include reduction or loss of the microvillar structures and cytoplasmic extroflession in the areas facing the lumen (asterisk). *n*=8 animals, collected from the wild, observed for each condition. Arrows indicate microvilli of epithelium; triangles indicate cytoplasmic extroflession. Scale bars: 5 µm.

Transmission electron microscopy (TEM) analysis of stomach sections of control (Fig. 1A) and animals treated (Fig. 1B-F) with 1% or 0.5% DSS overnight revealed significant morphological changes in the gut epithelium. In particular, the affected epithelial cells are characterized by a reduction or loss of microvillar structures and the distension of the plasma membranes into the area facing the lumen, resulting in cytoplasmic extroflession. This phenotype, which likely is due to disassembly of the cytoskeletal organization of the cells, is more severe in animals treated with 1% DSS (Fig. 1B-D) than in animals treated with 0.5% DSS (Fig. 1E,F).

**Fig. 2.**
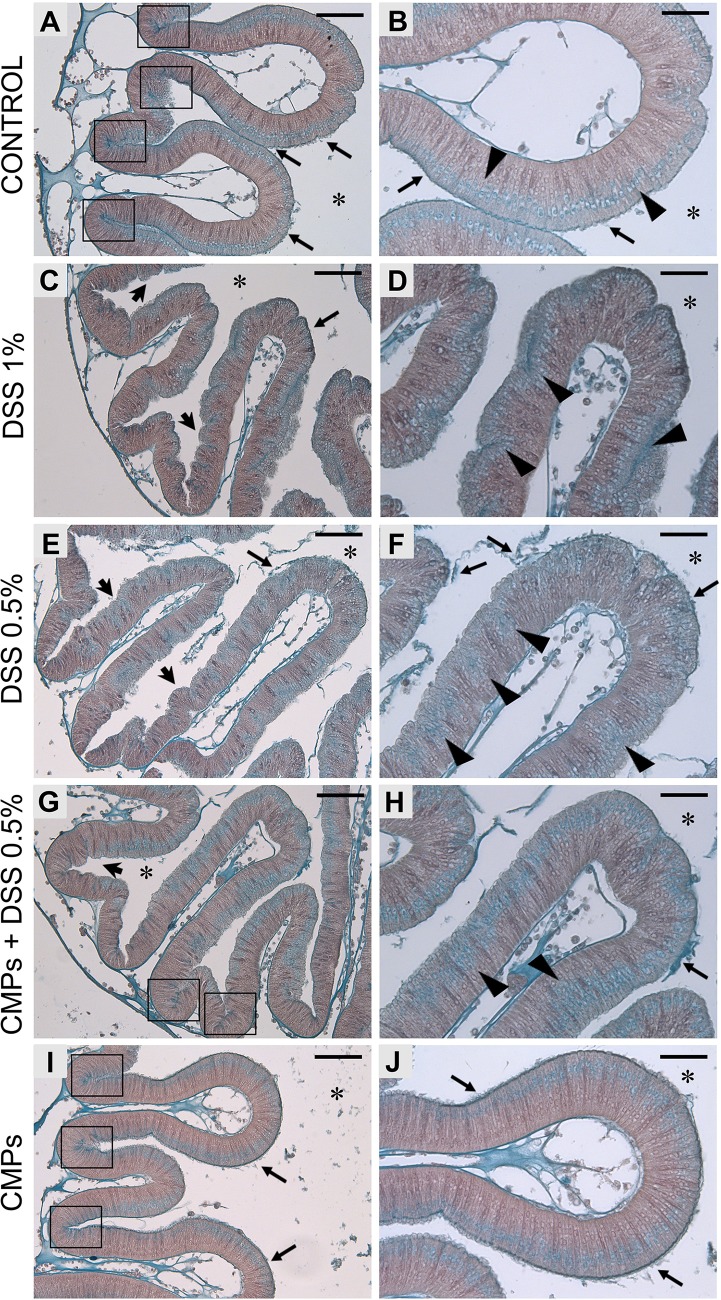
**Alcian Blue staining on stomach epithelium of adult *Ciona*.** Control animal, not DSS-treated (A,B), shows a thin and dense mucus layer on the surface of stomach epithelium (arrows). Glycoprotein-rich vesicles are strongly detected within the stomach grooves (A, delimited by rectangles) and spread along the epithelium, on the side closer to the gut lumen (B, triangles). Both 1% (C,D) and 0.5% (E,F) DSS-treated animals exhibit the epithelium characterized by the numerous furrows (arrowheads). In 1% DSS samples, a more intense blue staining is observed along the epithelium (C, arrow) and in cells that are more deeply located in the epithelial layer (D, triangles). Glycoprotein-rich granules are not detected in the grooves of stomach villi. In 0.5% DSS-treated samples, the mucus layer appears detached or loosely associated (E,F, arrows) and glycoprotein-rich granules are detected more deeply in the epithelium (F, triangles). The presence of CMPs during 0.5% DSS treatment reduced the formation of furrows on the epithelial surface (G, arrowhead), although a loosely associated mucus layer is still observed (H, arrow). Glycoprotein-rich granules are localized in the stomach grooves (G, rectangles) and less in the basal side of the epithelium (H, triangles). Stomach sections of animals treated with CMPs (I,J) reveal an epithelial morphology similar to that observed in control animals. *n*=6, animals observed for each condition. Asterisk indicates stomach lumen; rectangles, grooves of the stomach epithelium; black arrow, mucus layer; arrowhead, furrows; triangles, granules along the epithelium. Scale bars: (A,C,E,G,I) 50 µm; (B,D,F,H,J) 25 µm. Right images are magnification of the left images.

**Fig. 3.**
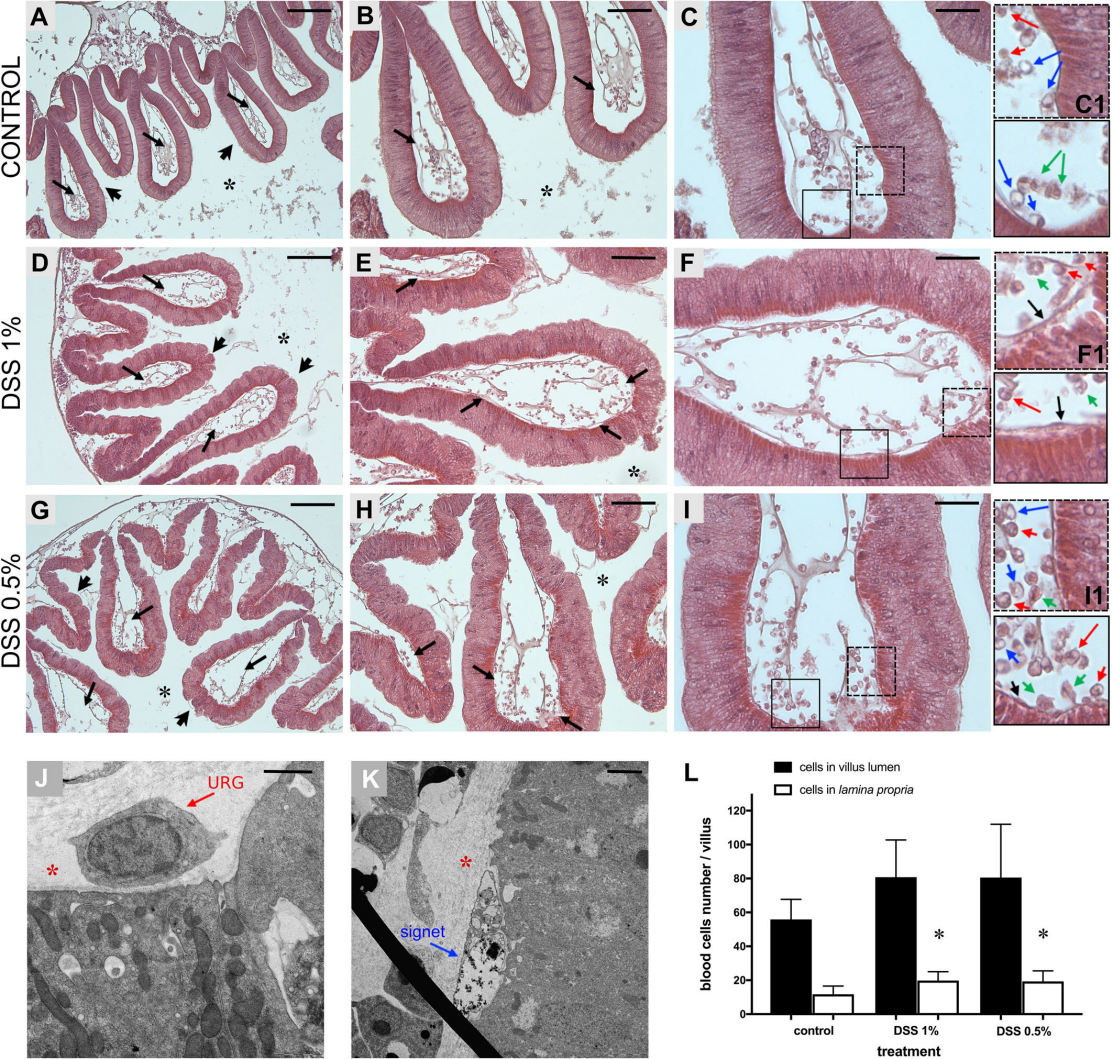
**Hemocytes of the stomach villi in animals treated with DSS.** Hematoxylin-eosin staining of stomach sections reveals a smooth continuous epithelium in control animals (A, arrowheads) and furrows-rich epithelia in DSS-treated samples (D,G, arrowheads). The lumen of stomach villi of control animals (A, arrows), 1% (D, arrows) and 0.5% (G, arrows) DSS-treated animals is populated by numerous hemocytes. A higher magnification of the villi reveals that the lamina propria of 1% (E, arrows; F) and 0.5% (H, arrows; I) DSS-treated animals is characterized by an increase in the number of blood cells, compared to controls (B, arrows; C). (C1,F1,I1) Magnification of the areas delimited by rectangles in C,F,I, respectively, highlights some of the cell type infiltrating the lamina propria and closer to the epithelium (red arrows, URG cells; blue arrows, signet cells; green arrows, amoebocytes; black arrows, cells in the basement membrane, adjacent to the epithelium). TEM confirms the presence of URG and signet cells closely associated with the basement membrane of stomach epithelium in DSS-treated samples (J,K). Red asterisk, lamina propria; black asterisk, stomach lumen. Scale bars: (A,D,G) 100 µm; (B,E,H) 50 µm; (C,F,I) 25 µm; (J) 1 µm and (K) 2 µm. (L) Abundance of hemocytes represented as the total cell numbers within the villus and infiltrated cells of the lamina propria, closer to the epithelium. Vertical bars represent the mean±s.d. of *n*=6, biological replicates for each condition. Statistical methods: one-way ANOVA. Asterisk indicates *P* value <0.05.

Histological observations reveal that after DSS treatment, the smoother continuous stomach epithelium (Figs 2A,B and 3A-C) is characterized by the formation of numerous furrows (Figs 2C-F and 3D-I) and changes to the production of mucus. In control animals, Alcian Blue staining shows a continuous thin mucus layer on the stomach epithelium and numerous cells containing glycoprotein-rich vesicles (Fig. 2A,B), which are mainly localized to the stomach grooves (Fig. 2A) and uniformly distributed along the (apical side of) epithelium, closer to the gut lumen (Fig. 2B). In 1% DSS-treated animals, an increase in mucus production is underscored by a more intense Alcian Blue staining of the epithelium, though not uniformly localized (Fig. 2C,D). In the stomach grooves, less glycoprotein-rich granules are detected and, along the epithelial layer, they are more deeply localized (Fig. 2C,D). This damage may reflect an effort to repair the epithelium. The lower dose treatment, 0.5% DSS, reveals a comparatively weaker effect on gut epithelium. A loosely associated mucus layer can be seen detaching from the epithelium (Fig. 2E), although an increase in mucus production is not suggested. A lower abundance of visible granules, more basally localized, can be observed in the stomach groove epithelial cells (Fig. 2E,F).

Additionally, light microscopic observation of these stomach sections suggests a relative increase in the number of hemocytes in the lamina propria, closer to the epithelium (Fig. 3). Cell counts confirm a significant increase in infiltrating cells within the lamina propria in both 1% and 0.5% DSS treatments; in contrast, an increase in the total number of hemocytes within the stomach villi lumen is not significant (Fig. 3L). TEM analysis further defines the hemocytes, particularly those found at the basal surface of the epithelium, as granular amoebocytes. The highest percentages of vacuolated cells observed at the TEM are indicated as univacuolar refractile granulocytes (URG) and signet cells (Fig. 3J,K). This type of general immunocyte infiltration is a characteristic feature of DSS-mediated experimental colitis in mammals (Dieleman et al., 1998; Okayasu et al., 1990; Perše and Cerar, 2012).

**Fig. 4.**
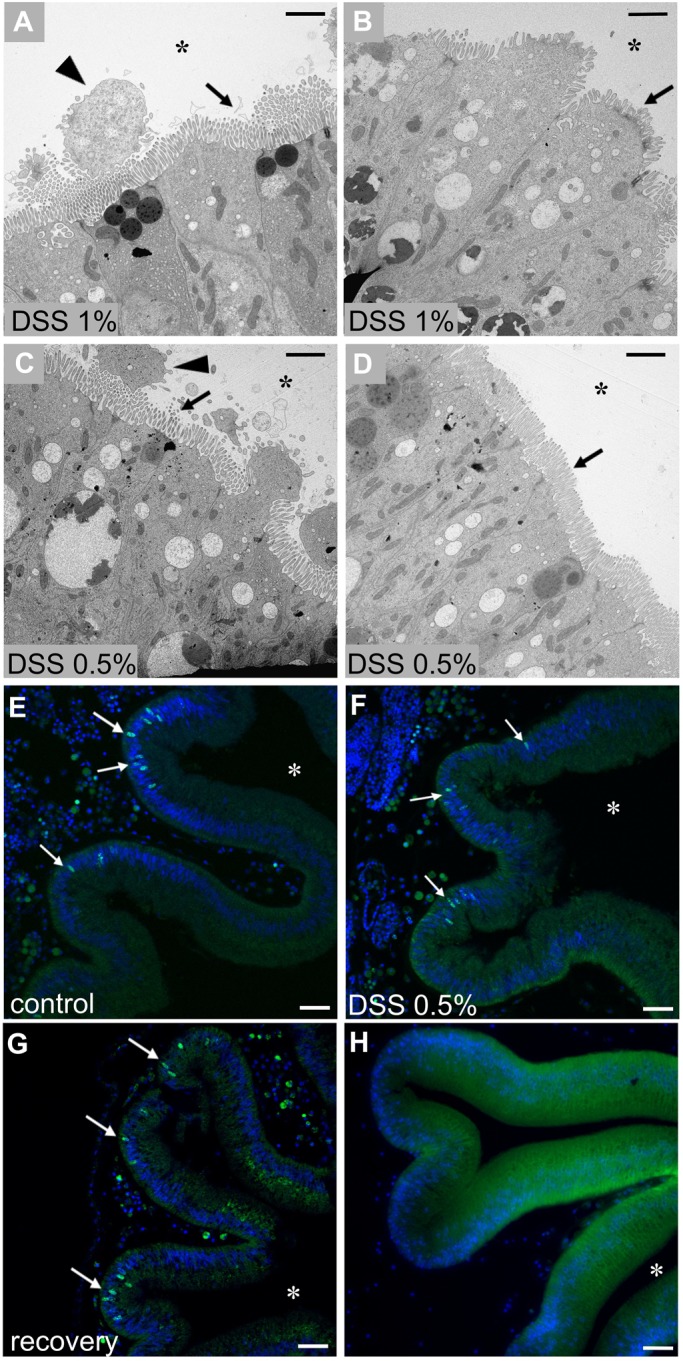
**Adult stomach epithelium of DSS-recovered animals.** TEM observation of the stomach epithelium one week after 1% (A,B) and 0.5% (C,D) DSS treatment. The stomach epithelium of recovered animals appears comparable to the controls, with typical microvilli morphology (arrows) and only some remnants of the damaged cells (triangles) in the stomach lumen. Proliferation activity of the epithelial cells (white arrows) in control (E), DSS 0.5% (F), and recovered (G) specimens. Control samples, not EdU treated (H). *n*=6 animals observed for each condition and technique. Asterisk indicates stomach lumen. Scale bars: (A-D) 4 µm; (E-H) 20 µm.

Further validation of DSS-induced colitis in *Ciona* has been obtained by recovery experiments. One week after the overnight treatment with either concentration of DSS, partial or almost complete recovery can be achieved (Fig. 4A-D). In these recovered specimens, the epithelium appears comparable to the controls, with a normal microvillar structure. Remnants of damaged cells in the process of being released into the stomach lumen (Fig. 4A,C) can be detected. The patterns of cell proliferation in stomach epithelium among control (Fig. 4E), DSS 0.5% (Fig. 4F) and ‘recovered’ animals (Fig. 4G) are indistinguishable.

**Fig. 5.**
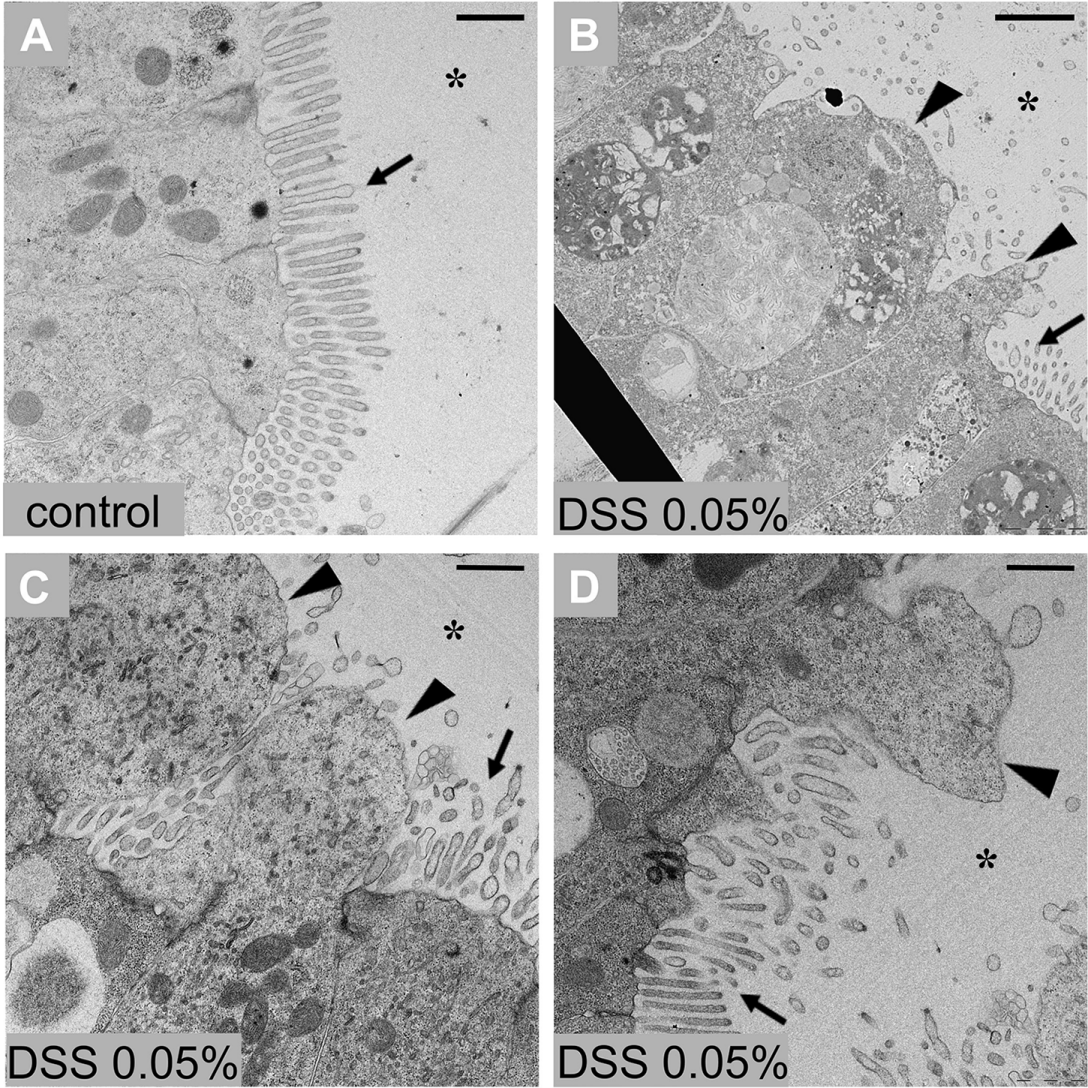
**Stomach epithelium of stage 7/8 ascidian juvenile after DSS treatment.** (A) Control epithelium. (B-D) Stomach epithelium 3 h after 0.05% DSS treatment shows altered ultrastructure in which the loss of microvilli (arrows) and cytoplasmic extroflessions (triangles) are evident. *n*=6 animals observed for condition. Asterisk indicates stomach lumen. Scale bars: 10 µm.

The effects of DSS also were observed in stage 7/8 of the 1st ascidian juveniles. Because of their delicate nature at this stage of development, it was necessary to use a lower concentration of DSS. Specifically, these animals were maintained in a solution of seawater containing 0.05% DSS for 3 h; viability was confirmed by observing the heartbeat under a stereomicroscope (Fig. S1F,G). The treated juveniles then were processed and analysed by TEM. Extensive loss of microvilli and the extroflessions of the cytoplasm were apparent among stomach epithelial cells (Fig. 5). Because very little is known about the abundance and nature of immunocyte development at this stage of metamorphosis, which is often met with a variety of technical challenges, their accumulation was not assessed in these treated juveniles.

### DSS effects innate immune gene expression at stage 7/8 of 1st ascidian juvenile

**Fig. 6.**
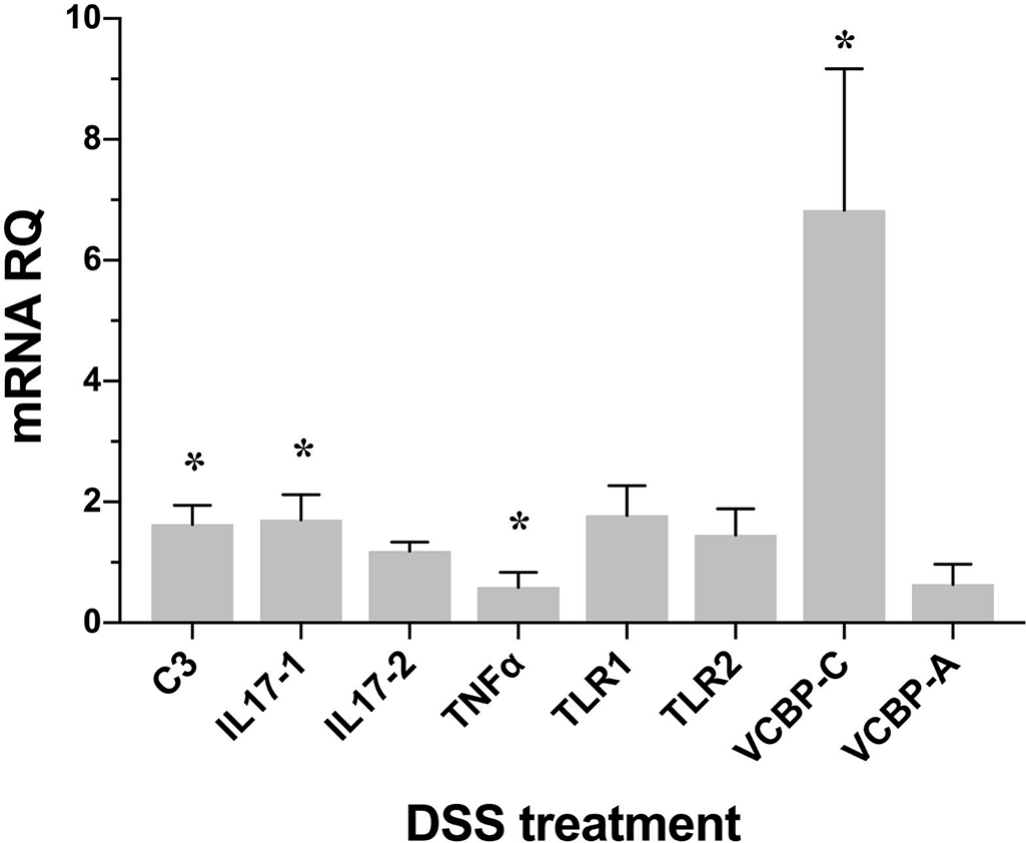
**Expression levels of immune genes following DSS treatment.** Changes in the expression of: *C3*, *IL-17-1*, *IL-17-2*, *TNFα*, *TLR1*, *TLR2*, *VCBP-A* and *VCBP-C* in stage 7/8 of the 1st ascidian juvenile treated with 0.05% DSS are measured by qPCR. Expression levels are reported as fold changes of relative RNA quantification (RQ) compared to the corresponding control samples. Vertical bars represent the mean±s.d. (*n*=3, biological replicates). Statistical methods: independent samples *t*-test. Asterisk indicates *P* value <0.05.

The expression of selected innate immune genes in the gut of *Ciona intestinalis*; including: tumor necrosis factor α (*TNFα*), the third component of the complement system *C3*, interleukins 17-1 (*IL-17-1*) and 17-2 (*IL-17-2*), the only two Toll-like receptors *TLR1* and *TLR2* identified in *Ciona*, as well as the immunoglobulin variable region-containing chitin binding proteins (VCBPs), *VCBP-A* and *-C* genes were characterized using qPCR. In order to avoid the high degree of interindividual variation, which is seen in freshly harvested (adult) animals, this experimental series was confined to stage 7/8 laboratory-rearing juveniles. The treatment with 0.05% DSS for 3 h, the amount of time required to induce morphological changes related to colitis, induces a sixfold increase of *VCBP-C* and a weak but significant increase in the expression of *C3* and *IL-17-1*. A weak but not significant increase of *TLR1*, *TLR2*, and *IL-17-2* also is seen. A significant decrease in the expression of *TNFα*, a usual marker of inflammation, was noted. The change in expression of *VCBP-A* is insignificant (Fig. 6).

### DSS treatment alters localization of *Ciona* gut-specific molecules

**Fig. 7.**
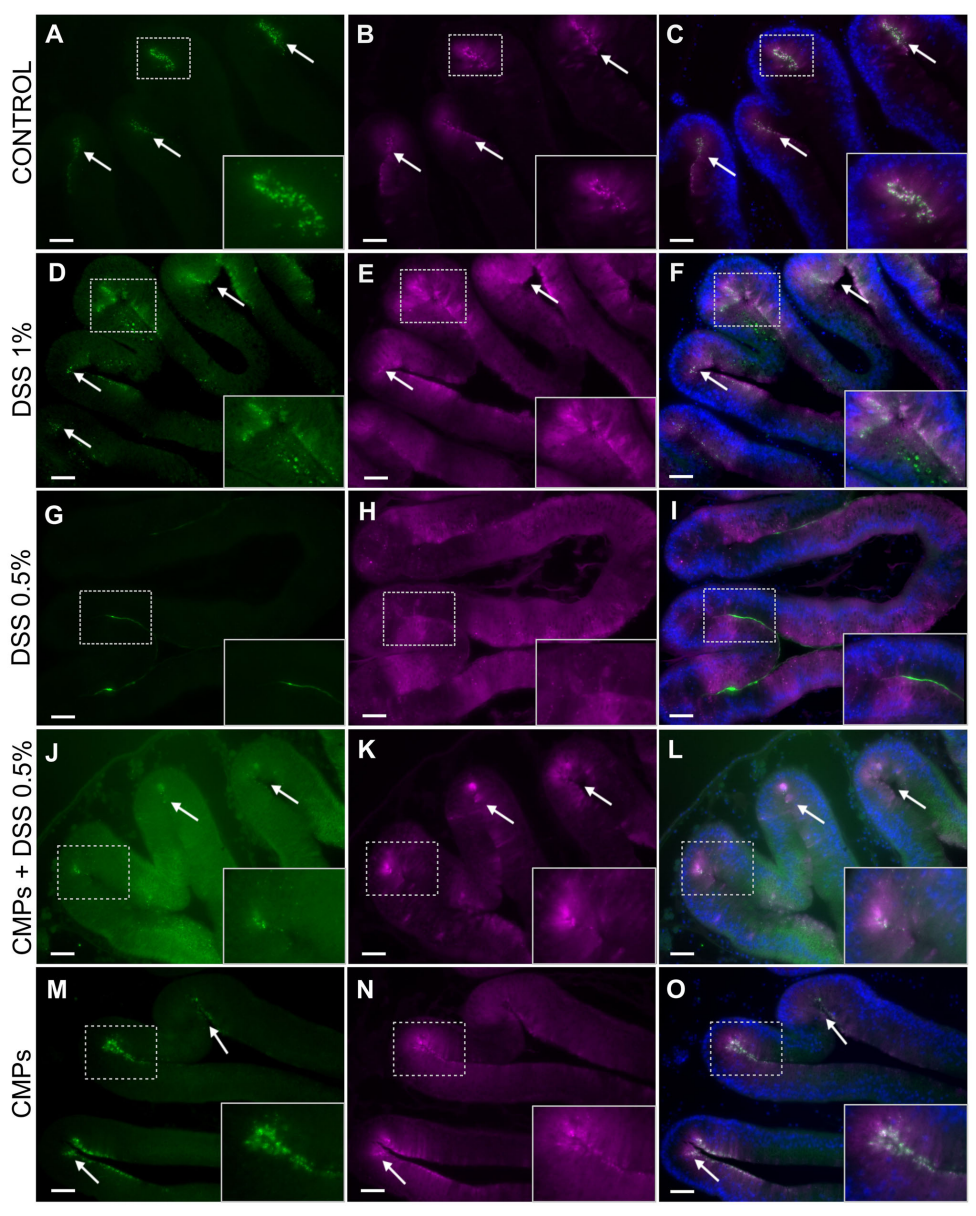
**VCBP-C and chitin localization in stomach sections of adult DSS- and CMP-treated animals.** In control animals, chitin (A, arrows) and VCBP-C (B, arrows) are detected in granules that predominantly co-localize (C, arrows) to the stomach grooves. In 1% DSS treated animals, chitin-rich granules are overexpressed in the crypts and along the epithelium (D, arrows); VCBP-C (E, arrows) production is increased and distributed evenly among the gut epithelium. Chitin and VCBP-C co-localization is observed in some region along gut epithelium (F, arrows). In 0.5% DSS-treated animals, chitin detection in the granules is reduced (G) and VCBP-C (H) no longer is detectable in granules at the epithelial crypts, but instead is localized along the epithelium. Co-localization of chitin and VCBP-C no longer is observed (I). In stomach sections of animals treated with 0.5% DSS and CMPs, fewer chitin granules are detected in stomach grooves (J, arrows), also where VCBP-C granules are observed (K, arrows), co-localization of these molecules can be observed in the same grooves (L, rectangle). In CMP-treated animals, chitin- (M, arrows) and VCBP-C-rich (*N*, arrows) granules are detected and co-localize (O, arrows) mainly in the stomach grooves, as observed in control untreated animals. Animals observed, *n*=8 for CTR, DSS 1%, DSS 0.5% conditions; *n*=6 for CMPs and CMPs/DSS 0.5% conditions. Inserts, magnification of the area delimitated by rectangles. Scale bars: 100 µm.

Chitin is a structural component of the epithelium-associated mucus of the gut in *Ciona intestinalis* (Dishaw et al., 2016). Products of the VCBP gene family are expressed specifically in the gut during morphogenesis (Liberti et al., 2014) and localize to the gut epithelium of the adult animals (Dishaw et al., 2011; Liberti et al., 2014). Among the VCBPs, VCBP-C is secreted into the gut lumen where it binds bacteria (Dishaw et al., 2011) and co-localizes with chitin fibers that are endogenously expressed and secreted together with the gut mucus from the earliest stages of digestive tract development (Dishaw et al., 2016). In control samples, VCBP-C and chitin can be detected (prior to release) in granules that co-localize primarily to the grooves of stomach; the base of the epithelial grooves is the primary location of synthesis for these secreted molecules (Fig. 7A-C). Adult animals treated with DSS exhibit alterations in both the expression and localization of both VCBP-C and chitin. In 1% DSS-treated animals, VCBP-C production is increased and it is distributed uniformly throughout the gut epithelium. Chitin-rich granules are overexpressed in the crypts and along the epithelium. VCBP-C and chitin can still be seen to co-localize in the grooves of stomach at this concentration of DSS (Fig. 7D-F). Among animals treated with 0.5% DSS, VCBP-C no longer can be detected in granules at the epithelial crypts, but instead is localized along the epithelium and strongly at the borders of the villi. Chitin detection in granules also is reduced, reflecting the possibility that it (along with mucus) may be released into the lumen to counter a compromised barrier (Fig. 7G-I).

### CMPs protect *Ciona* gut epithelium from the DSS treatment

**Fig. 8.**
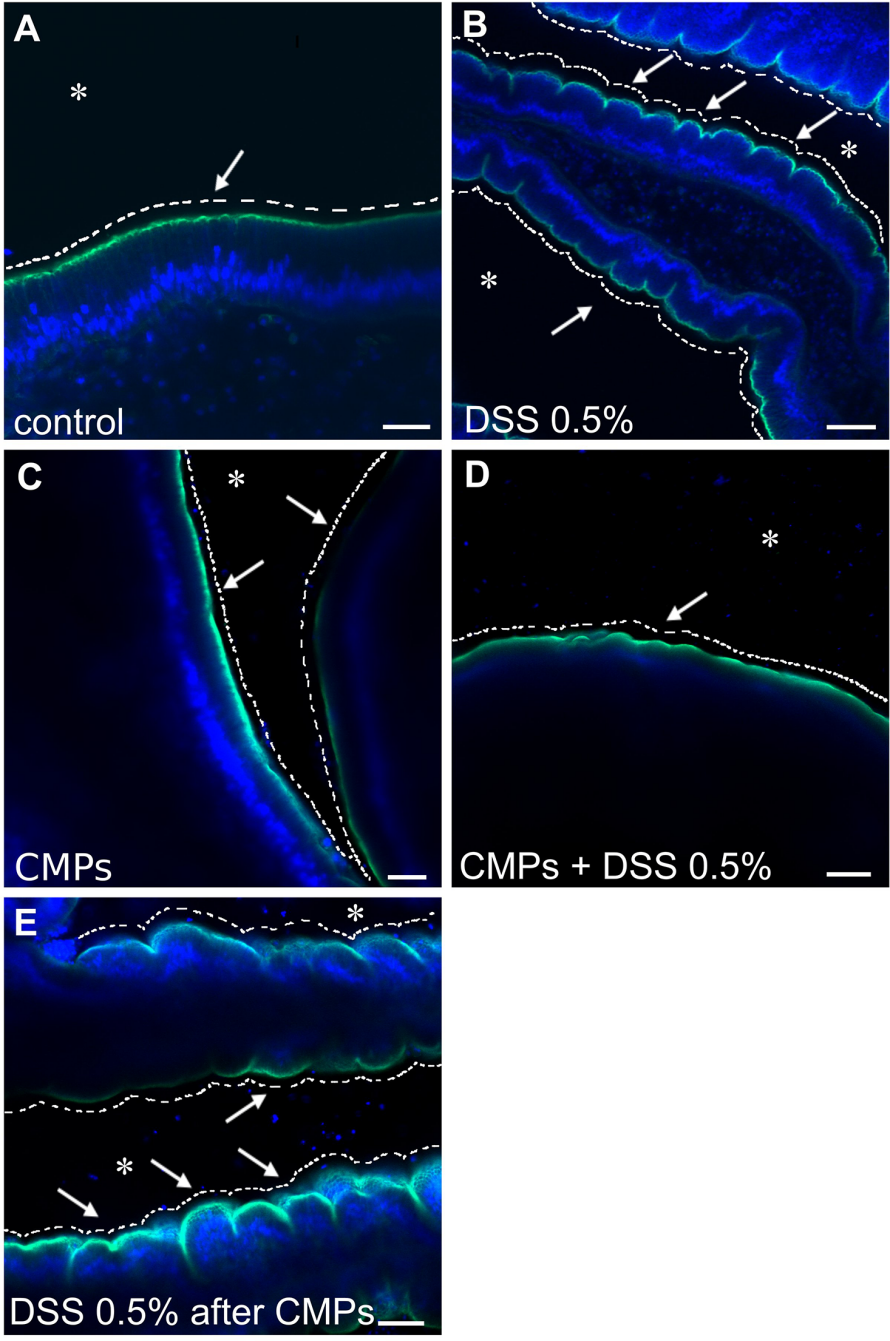
***In vitro* effect of CMPs on adult DSS-treated stomach explants.** The smooth continuous layer of the control stomach epithelium (A, arrow) appears as a festoon structure in the stomach of the DSS-treated animals (B, arrows). CMPs, which had no direct effect on the morphology of the epithelium (C, arrows), reduce the effects of the DSS treatment if maintained in the incubation medium; the surface of the epithelium instead remains smooth (D, arrow). Removal of the CMPs from the DSS-containing medium resulted in a phenotype comparable to that of the DSS-treated samples (E, arrows). Asterisk indicates stomach lumen. White dashed lines have been drawn to highlight the surface morphology of the epithelium, smooth or furrows characterized. Green staining, Alexa Fluor 488-Phalloidin; blue staining, DAPI. *n*=8 animals observed for each condition. Scale bars: (A) 20 µm; (B,C) 40 µm; (D) 100 µm and (E) 50 µm.

In order to determine if CMPs reduce or prevent DSS-mediated colitis and associated inflammation, experiments were carried out initially on dissected stomach pieces to verify the direct effect of CMPs on the gut. Samples were analyzed by confocal microscopy after staining with Alexa-Fluor 488 phalloidin, which serves as a marker of the cytoskeletal structure underlying the plasma membrane, and is informative of changes to the overall morphology of stomach epithelium. In control, untreated samples the apical surface of the epithelium (facing the lumen) appears as a smooth continuous layer with sparse scattered furrows (Fig. 8A); in DSS-treated animals, the apical surface of the stomach possesses festoon-like structures due to a significant increase in the number of furrows (Fig. 8B). CMPs, which in separate control experiments did not alter morphology of the epithelium (Fig. 8C), reduce the effects of the DSS treatment (Fig. 8D) when the CMPs were maintained in the incubation medium throughout the experimental treatment with DSS. Removal of CMPs before DSS treatment produced a phenotype comparable to that of the DSS-treated experimental samples (Fig. 8E). Identical results were obtained when CMPs were administered to adult live animals that were treated with DSS (Fig. S2).

Protective effects of CMPs on gut epithelium during DSS treatment has also been investigated on gut sections. Both Alcian Blue staining, for the observation of epithelial morphology and mucus damage, and immunofluorescence experiments, for the localization of *Ciona* specific molecules such as VCBP-C and chitin, have been performed. Alcian Blue staining reveals a reduction in epithelial damage induced by the compound when CMPs are present during DSS treatment. Specifically, less furrows are observed along the stomach epithelium and more glycoprotein-rich granules are detected in the stomach grooves (Fig. 2G-H) compared to samples that are only DSS-treated (Fig. 2E,F). Also, glycoprotein-rich granules are more uniformly distributed along the apical side of the epithelial layer; however, as in DSS treatments, some areas are found to possess granules more deeply localized in the epithelium (Fig. 2H). Mucus layer detachment from the epithelium can still be observed (Fig. 2G,H). In CMP-treated animals, the gut epithelium demonstrates morphological similarities to control samples, in mucus (Fig. 2I,J) and VCBP-C and chitin granule co-localization within stomach grooves (Fig. 7M-O). Adult animals treated with CMPs and DSS exhibit a decrease of both VCBP-C and chitin granules in stomach grooves (Fig. 7J-L); however, neither overexpression nor mislocalization has been observed as in DSS-treated samples (Fig. 7D-I). Collectively these data suggest a weaker effect of DSS compound on the gut epithelium in presence of CMPs.

## DISCUSSION

It stands to reason that animals have evolved multifaceted approaches at the mucus-rich surface of gut epithelia as a first-line of barrier defense in response to colonizing microorganisms (Lievin-Le Moal and Servin, 2006). In particular, host-microbe dynamics at gut epithelial surfaces are governed primarily by innate immune responses which, when misguided, can lead to inflammatory pathologies like IBD (Baumgart et al., 2009; Hampe et al., 2001; Hart et al., 2005; Ogura et al., 2001; Vijay-Kumar et al., 2007a,b). Beyond microbial dysbiosis, a combination of factors including environmental stressors and genetic predisposition, e.g. erroneous or deficient immune responses, are recognized in the onset of IBD, to eliminate chronic, relapsing, inflammatory disorders that affect the colon (as in ulcerative colitis) and other parts of the gastrointestinal tract (as in Crohn's disease), severely impacting human wellness (Baumgart and Carding, 2007; Khor et al., 2011).

A variety of animal systems have been developed to study experimentally induced colitis (e.g. utilizing chemicals such as DSS) and to investigate the onset and development of pathogenesis and better define the complexity of immune responses, improve our understanding of the role of resident microbiota, as well as host genetic predisposition (Anderson et al., 2011; Franke et al., 2010) and environmental influences, such as stress (Aamodt et al., 2008). DSS*-*treated *Ciona intestinalis* are shown here to develop a colitis-like phenotype similar to what has been described in other systems; however, unlike traditional approaches in which animals are exposed to the DSS only when drinking water (i.e. intermittent exposure), *Ciona*, as a siphon-feeding marine organism, experiences continuous exposure (i.e. immersion treatment). Over a concentration range between 0.5% and 1% DSS, varying degrees of sustained, and severe, colitis-like phenotypes are induced; a reduced treatment time and lower exposure dose can sustain the desired pathologic conditions.

In a mouse model, the histological changes associated with DSS-induced colitis have been classified as acute (early) or chronic (advanced) (Okayasu et al., 1990; Perše and Cerar, 2012). The former condition is associated with mucin depletion, epithelial degeneration and necrosis; the latter phase also is characterized by an infiltration of neutrophils into the lamina propria and submucosa, the formation of cryptitis, and crypt abscesses (Okayasu et al., 1990; Perše and Cerar, 2012). Distinct inflammatory profiles have been described in the two phases of colitis. An increase in the expression of *TNFα*, *IL-6* and *IL-17* is associated with acute colitis, whereas decreased expression is reported in chronic conditions (Alex et al., 2009). In *Ciona*, DSS treatment induces epithelial degeneration characterized by reduction or complete loss of microvilli structure and plasma membrane distension at the apical side of the epithelium, as well as an increase in the number of immunocytes at the basal side within the lamina propria. A decrease in *TNFα*, along with a slight increase in *IL-17* expression, is seen. If treatment is interrupted, complete recovery can be achieved within one week. In mammalian DSS colitis, healing of the epithelial barrier is regulated in part by an increase in the natural turnover rate of the epithelial cells, a process which itself is controlled by factors such as diet and microbiota, host transcription factors and pro-inflammatory cytokines (Chassaing et al., 2014). Repair of the gut epithelium in *Ciona* does not coincide with an increase in the natural turnover rate of the gut epithelial cells. DSS-induced colitis in the *Ciona* model is unique in that it reflects features of both acute and chronic conditions observed in mammals.

Pathogen recognition receptors play important roles not only in recognition of the host-associated microbiota, but also in the regulation of inflammation, protection from damage and in the healing of damaged barriers (Cario, 2010; Fukata and Arditi, 2013; Huebener and Schwabe, 2013; Rose et al., 2012b). As a major sensor of the innate immune system, Toll-like receptors (TLRs) are able to recognize a broad range of microorganisms and trigger an immediate inflammatory response. In mouse gut, the interaction between TLRs and microbes plays a critical role in the maintenance of normal homeostasis (Rakoff-Nahoum et al., 2004). In the mouse colitis model, TLRs have been suggested to serve a protective role (Lee et al., 2006; Rakoff-Nahoum et al., 2004; Vijay-Kumar et al., 2007b). In *Ciona*, however, the TLRs are up-regulated only slightly during DSS treatment. *Ciona* possesses only two TLRs (CiTLR 1 and 2), which together with VCBPs, are expressed primarily in the digestive tract (Dishaw et al., 2011; Liberti et al., 2014; Sasaki et al., 2009). VCBPs, which are found in some protochordate (Cannon et al., 2002; Cannon et al., 2004; Dishaw et al., 2011, 2008), consist of two immunoglobulin (Ig) V-type domains and a chitin-binding domain (CBD), recognize bacteria by way of the Ig domains and bind to chitin in the gut via the CBD. *Ciona* VCBP-C functions as an opsonin by increasing the phagocytic activity of amoebocytes (Dishaw et al., 2011), and has a role in modulating the onset of bacterial biofilms (Dishaw et al., 2016). VCBP-C binds bacteria in the gut (Dishaw et al., 2011, 2016) and associates with an extensive network of chitin fibers interwoven into the epithelial-associated mucus, suggesting a possible function in modulating biofilm formation adherent to the epithelial surfaces (Dishaw et al., 2016). During DSS treatment, *VCBP-C* exhibits an approximate sixfold increase in relative expression (Fig. 6).

Besides its known chemical toxicity towards epithelial cells, DSS treatment leads to increased permeability and disruption of tight junctions (Kitajima et al., 1999; Poritz et al., 2007; Turner, 2009) and mediates changes to the biophysical structure of mucus barriers, contributing to bacterial colonization of inner mucus layers (Dicksved et al., 2012; Johansson et al., 2010) that normally are devoid of bacteria (Johansson et al., 2008). Bacteria that penetrate the inner mucus layer come into contact with host epithelium and trigger an inflammatory reaction (Johansson et al., 2010). It is not entirely clear how mucus is structured in *Ciona*; however, the gut epithelium is layered with chitin-rich mucus, with two distinct layers evident in some areas (Dishaw et al., 2016), similar to that described in mammals (Johansson et al., 2008). Genes encoding mucin-related proteins can be identified in the *Ciona* genome, as well as in other ‘lower’ metazoans (Lang et al., 2007), and their homology to mammalian mucins is unclear.

The integrity of mucus layers in *Ciona* has been monitored with a biological stain such as Alcian Blue. Qualitative disruption and changes to mucus encompass DSS treatment in various model systems (Chassaing et al., 2014; Oehlers et al., 2012, 2013) as is seen here in *Ciona*, in which the mucin barriers are chitin-rich. The observed disruption likely facilitates microbial interactions with the epithelial surface. Furthermore, depletion of the chitin-rich mucus, along with epithelial damage and exposure to microbes, may contribute to the rapid increase in VCBP-C expression, which is confined to the grooves of stomach epithelial crypts (Liberti et al., 2014) in which granules (or other vacuole-like compartments) containing chitin also are detectable in normal healthy animals. Following DSS treatment, both VCBP-C and chitin-positive epithelial cells can be detected along the length of the gut epithelium, and at the borders of the crypts. The lack of expression of VCBP-C at the base of the crypts could be associated with change or damage to the goblet-like cells that become damaged by chemical induction of colitis (Chassaing et al., 2014), as also has been observed here by Alcian Blue staining (Fig. 2). Chitin production is reduced at lower doses of DSS. Collectively, these data suggest that DSS treatment influences the synthesis and/or release of chitin, which likely is exuded with mucus, suggesting a correlation with the repair or reinforcement of the damaged mucus barriers. The marked increase in expression of VCBP-C may by coupled to chitin and/or mucus production, along with recognition and binding to bacteria and subsequent biofilm formation that is affected by the DSS treatment.

Based on the pathological similarities and responses to DSS treatment, it is tempting to speculate that the role of VCBPs in the gut of *Ciona* may be analogous to that of secretory immunoglobulin A (SIgA) in mammals (Dishaw et al., 2014a; Liberti et al., 2015). Both SIgA and VCBP-C may modulate settlement of bacteria at the epithelial surface in the gut lumen (Dishaw et al., 2016). SIgA molecules have been shown to identify commensal bacteria that preferentially affect IBD susceptibility (Palm et al., 2014); specifically, a high-affinity SIgA modulates commensal microbial composition and provides a notable therapeutic effect in the mouse colitis model (Okai et al., 2016). Additional studies on differential VCBP-C binding to the *Ciona* gut bacteria and the role that distinct bacterial taxa have in shaping gut microbial communities, under both physiological and pathologic conditions, potentially can help define the complex roles for immune effector such as secreted, Ig-related immune molecules, in barrier defenses.

Although the expression of endogenous chitin is lacking in mammals (e.g. chitin synthase genes were lost in ancestral taxa) (Tang et al., 2015; Zakrzewski et al., 2014), the phylogenetic distribution of chitin-rich mucus layer coating gut epithelial cells is far broader among animals than considered previously (Tang et al., 2015). Administration of CMPs improves the outcome of experimentally induced colitis and can modulate immune responses during intestinal inflammation (Nagatani et al., 2012). Chitin is known to modulate various aspects of both innate and adaptive immunity, reflecting the conservation of ancient recognition pathways likely tied to the recognition of fungi and/or ectoparasites (Da Silva et al., 2009, 2008; Elieh Ali Komi et al., 2017; Lee et al., 2008; Reese et al., 2007; Wagener et al., 2017). CMP treatment enhances intestinal epithelial function and influences the composition of the gut microbiome in mammals (Nagatani et al., 2012). As is seen here in *Ciona*, whereas the mechanism of action of CMPs is unclear, their function may not be limited to only enhancing barriers, but immune signaling molecules such as cytokines also are affected (Nagatani et al., 2012). The merit of a protochordate model system that retain basic characteristics of other vertebrate model can inform us of phylogenetically ancient signals, which modulate homeostasis and protect animals from colitis-like inflammatory processes.

The new paradigm of recognizing animals as metaorganisms represents a strong imperative for defining host-microbiota interactions in pathogenic as well as under healthy circumstances. *Ciona intestinalis*, whose gut microbiota is known (Dishaw et al., 2014b), with many isolates now in culture (L.J.D., unpublished), and recently established as a germ-free system (Leigh et al., 2016), clearly is an informative model for defining the host-microbial dynamics at epithelial borders. The *Ciona* gut is simpler than mammalian model systems, lacks contributions from adaptive immunity and a complex network of gut associated lymphoid tissues, yet retains the ancient feature of chitin integration into mucosal barriers. The studies reported here further underscore the merits of the *Ciona* model by demonstrating that DSS treatment mediates a similar and reproducible pathological phenotype involving colitis-like inflammation and damage. Because innate immunity is the first line of response in vertebrates, and the only form of immunity in all other animals, *Ciona*, which permits the study of innate immunity in isolation, may help us determine how innate immunity modulates recovery from colitis and the re-establishment of homeostasis.

## MATERIALS AND METHODS

### Ethics statement

The research described herein was performed on *Ciona intestinalis* (subtype A, more recently recognized as *Ciona robusta*), a marine invertebrate collected in the Gulf of Napoli (Italy) or in San Diego, CA (M-Rep), in locations that are not privately owned nor protected in any way, according to the authorization of Marina Mercantile (DPR 1639/68, 09/19/1980, confirmed on 01/10/2000). The study did not involve mammalian or vertebrate subjects, or endangered or protected species, and was carried out in strict accordance with European (Directive 2010/63) and Italian (Decreto Legislativo n. 116/1992) legislation for the care and use of animals for scientific purposes. *Ciona* is considered an invasive species and is not regulated or protected by environmental agencies in the USA or Italy. The collection services contracted in this study maintain current permits and licenses for collection and distribution of marine invertebrates to academic institutions; special permission was not required to collect *Ciona*. Handling of live animals was in accordance with the guidelines of our academic institutions. Animals were recovered and brought to the laboratory alive and maintained in clean water with aeration. In accordance with general animal protocols, the least number of animals required per experiment were utilized. Animal waste products were disposed of appropriately.

### Treatment with DSS

Adult *Ciona* were immersed in FSW containing 0.5% or 1% DSS (40 kDA, TdB, Uppsala, Sweden) and maintained at 18°C overnight; viability was determined by verifying the response to external stimuli, such as gentle poking with a Pasteur pipette and the presence of a heartbeat. Animals were sacrificed and stomach samples were collected. In recovery experiments, DSS-treated animals were transferred to circulating seawater for one week. Stomachs from controls, as well as animals treated with DSS and animals recovered from DSS treatment were fixed and processed for TEM or other histology procedures (see below).

*Ciona* stage 7/8 juveniles, developed from a single batch of gametes as described previously (Liberti et al., 2014), were divided into two samples, and maintained as a control or treated with 0.05% DSS for 3 h. These experimental conditions, which produced the same phenotype(s) observed in adults treated with 1% DSS, were refined to minimize the high mortality observed in higher dose DSS treatment of the younger, more experimentally labile, 7/8 juveniles.

### Chitin microparticles treatment

Groups of four wild-collected animals were incubated in 750 ml of FSW containing 15 mg/l of CMPs [1-10 µm in size (Nagatani et al., 2012)] for 6 h. Animals were then incubated overnight in 750 ml fresh FSW containing 15 mg/l of CMPs and 0.5% DSS at room temperature. All incubations were performed on a magnetic stirrer in order to maintain CMPs in suspension. Three control groups consisting of untreated, CMP-treated and DSS-treated animals were run in parallel. Animals were then moved to fresh FSW, and stomachs were dissected and fixed in methanol-Carnoy's fixative for histology techniques (see below) or in 4% paraformaldehyde, 50 mM MOPS and 500 mM NaCl, for 18 h at 4°C. These latter samples were washed three times in PBS for 10 min and processed for confocal microscopy.

For CMP treatment of explants, stomachs were dissected, cut longitudinally, in order to expose the lumen, and transferred to 12-well plates containing 2 ml FSW per well. Following 30 min incubation with 3 µg/ml CMPs and mild rotatory mixing, samples were incubated in fresh FSW containing CMPs at the same concentration and 0.5% DSS for 30 min. Specimens were fixed as described above and processed for confocal microscopy. Three controls consisting of untreated, CMP-treated and DSS-treated stomachs were run in parallel.

### Cell proliferation assay

Proliferation of stomach cells was evaluated based on the incorporation of 5-ethynyl-2′-deoxyuridine (EdU, Invitrogen) into DNA during active DNA synthesis. Adult *Ciona* were immersed in FSW containing 0.5% DSS and maintained at 18°C overnight. At the end of the incubation, some DSS-treated animals were immediately processed for EdU assay, others were transferred in fresh FSW for recovery experiment and used one week later for proliferation assay. The EdU assays were performed as following: overnight DSS-treated or recovery animals were immersed for 4 h in FSW containing 400 µM EdU, subsequently stomachs were dissected and fixed in 4% paraformaldehyde in FSW overnight at 4°C. Samples were dehydrated and embedded in paraffin and 7 µm sections were collected onto Superfrost Plus slides (Thermo Scientific). Controls, both EdU and no-EdU treatment, were run in parallel.

Following dewaxing and re-hydration of tissue sections, EdU-positive cells were detected using Click-iT^®^ EdU Imaging Kits (Invitrogen) according to manufacturer instructions. DAPI was used for DNA staining. Slides were observed under a confocal microscope LSM 510 META (Zeiss) and images were acquired employing Zeiss LSM Image Browser.

### Adult stomach and juvenile samples for observations at ultrastructural level

Stomachs from adult and juvenile animals were fixed overnight at 4°C in a mixture of 2.5% glutaraldehyde, 2% paraformaldehyde and 100 mM sodium cacodylate in FSW. After 18 h, samples were rinsed 6 times for 10 min in 100 mM sodium cacodylate in seawater and post-fixed in a solution of 1% osmium, 4% sucrose, 100 mM sodium cacodylate in ddH_2_O for 1 h at room temperature. Samples were washed in 100 mM sodium cacodylate in ddH_2_O and then dehydrated through a graded alcohol concentration series that, included two successive 15 min washes with 30%, 50%, 70% and 90% ethanol, and three successive 15 min washes with 100% ethanol. After dehydration, samples were embedded in EPON resin and 60 nm ultrathin sections were collected on 200 mesh nickel grids. Sections were counterstained with uranyl acetate (20 min) and lead citrate (40 s) and observed using a Leo 912 (Zeiss) TEM.

### Histology and immunofluorescence on gut sections

Stomachs from control animals, animals treated with 1% or 0.5% DSS and animals treated with CMPs, with or without 0.5% DSS, were fixed in methanol-Carnoy's fixative and embedded in paraffin as previously described (Johansson and Hansson, 2012). Immunofluorescence staining with specific anti-VCBP-C antibody (validated in Dishaw et al., 2011; Liberti et al., 2014) and recombinant human IgG1 Fc-CBD-C probe (validated in Dishaw et al., 2016) was performed as specified previously (Dishaw et al., 2016). Alcian Blue protocol was performed as described previously (Dishaw et al., 2016) with the modification of an overnight staining in Alcian Blue solution. Hematoxylin was used for nuclei staining.

Hematoxylin-eosin staining was performed as standard protocol. Briefly, after dewaxing and rehydration, sections were rinsed in ddH_2_O and the nuclei were stained with hematoxylin (Fisher, #CS401-4D) for 4 min. Sections were rinsed first in running tap water, then in Scott's tap water, and again in running tap water, before staining with Eosin-Y (Sigma, #HT110280) for 30 s. Sections were then dehydrated, cleared and mounted.

### Hemocytes cell quantification on gut sections

Stomach sections, stained by standard hematoxylin-eosin protocol, were observed at the light microscope. For each sample, ten random stomach villi were acquired at 400× magnification (as in Fig. 3C,F,I), and six different samples were analysed for each condition. The total number of the cells in each villus and cells in the lamina propria, closer to the basal membrane of the epithelium, were counted. The result represents the average of the blood cells counted in the ten villi. The average for all samples was used in calculating the average number of blood cells for each condition. The significance of the data obtained has be determined using one-way ANOVA statistical test, performed with PRISM software (www.graphpad.com/scientific-software/prism/).

### Confocal microscopic analysis of CMP-treated samples

Fixed CMP-treated samples were washed three times in PBS containing 0.7% Triton for 10 min, three times in PBS for 10 min and equilibrated in PBS containing 1% BSA. Samples were then incubated for 30 min in PBS/1% BSA containing 165 nM Alexa Fluor 488 phalloidin (Life Technologies #A12379). After incubation, samples were washed four times for 10 min in PBS and counterstained with 4′,6-diamidino-2-phenylindole (DAPI) when needed. Samples were observed under a LSM 510 META (Zeiss) confocal microscope and images were acquired employing Zeiss LSM Image Browser.

### Real-time PCR

Total RNA was extracted from stage 7/8 juveniles with SV total RNA isolation system (Promega) according to the manufacturer's instructions. Oligo(dT)-primed single-stranded cDNA was synthesized from 1µg of total RNA employing the SuperScript™ III First-Strand Synthesis System for RT-PCR (Invitrogen).

Gene expression was analyzed by quantitative real-time PCR (qPCR) on cDNA from control and 0.05% DSS treated juveniles. Primer sequences of the genes examined are listed in Table S1. *Ciona* cytoskeletal actin (*act*) was used as a reference for internal standardization. The amplification efficiency of each primer set was assessed employing 10-fold serial dilution of the cDNA of the 1st ascidian juvenile 7/8 stage. qPCR was performed according to the manufacturer's recommendation with the FastStart SYBR Green Master (Roche). A primer concentration of 0.28 nM, 1 µl of cDNA (diluted 1:10), denaturation at 95°C for 10 min and 40 amplification cycles (95°C for 15 s and 60°C for 1 min) were employed. Reaction of each sample was performed in triplicate on three different samples. Data were analysed with Vii™ 7 Real-Time PCR software (Life Technologies), and quantified with the comparative *C_t_* method (2^−ΔΔ*Ct*^) based on Ct values in order to calculate the relative mRNA expression level. The expression levels of the selected genes were evaluated in number-fold increase relative to the corresponding control samples. The significance of the relative ΔΔCt of each group (biological replicates *n*=3), compared to the controls, was determined using ‘independent samples *t*-test’. The statistical analyses were performed using PRISM software.

## Supplementary Material

Supplementary information
