# Supplementary material for: Chitin protects the gut epithelial barrier in a protochordate model of DSS-induced colitis
Source: Biol Open. 2017 Dec 8;7(1):bio029355. doi: 10.1242/bio.029355 (PMC5827265; doi:10.1242/bio.029355)
Supplement: Supplementary information [file biolopen-7-029355-s1.pdf]

Acronym	Gene name	GeneBank acc. no	Oligonucleotide (5'-3')	Amplicon (bp).	Slope	R^2	*E*
Ci-TLR1	Toll-like receptor1	AB495261	F cgtagccaatagctggacaa<br>R agtcgttcaacaccaaccaa	135	-3.08	0.982	100%
Ci-TLR2	Toll-like receptor2	AB495262	F aatgtgcattcacgagcgtg<br>R acgtcgagatgtgctgatgc	82	-2.17	0.935	100%
IL-17-1	Interleukin 17-1	NM_001129875	F acatgtccagcagacgagga<br>R caaccaacggtaatggtttg	127	-3.49	0.989	94%
IL-17-2	Interleukin 17-2	NM_001129874	F cgggtgcattgcttctagt<br>R cacgcaggtacagcctattg	145	-3.45	0.996	95%
TNFα	tumor necrosis factor alpha	NM_001128107	F caaatggaccgccattaaa<br>R cctgtttccgtgttccaaga	64	-3.33	0.975	100%
C3-1	complement component c3	NM_001032512	F agcgaaagatccattggtac<br>R gcaattcctggttccacagt	174	-3.36	0.997	99%
VCBP-A	Variable region-containing chitin-binding protein A	HQ324141	F atttgatacagtgacgtgg<br>R cggtagtgggaatatgaa	188	-2.84	0.979	100%
VCBP-C	Variable region-containing chitin-binding protein C	HQ324151	F agaccaacgccaacacagta<br>R cccatacattgcagcatttc	144	-3.16	0.996	100%
act	cytoskeletal actin	AJ297725	F cccaaatcatgttcgaaacc<br>R acaccatcaccactgtcgaa	118	-3.41	0.992	97%

Table S1. Primer sequences and efficiency of *Ciona* genes analyzed in qPCR experiments.

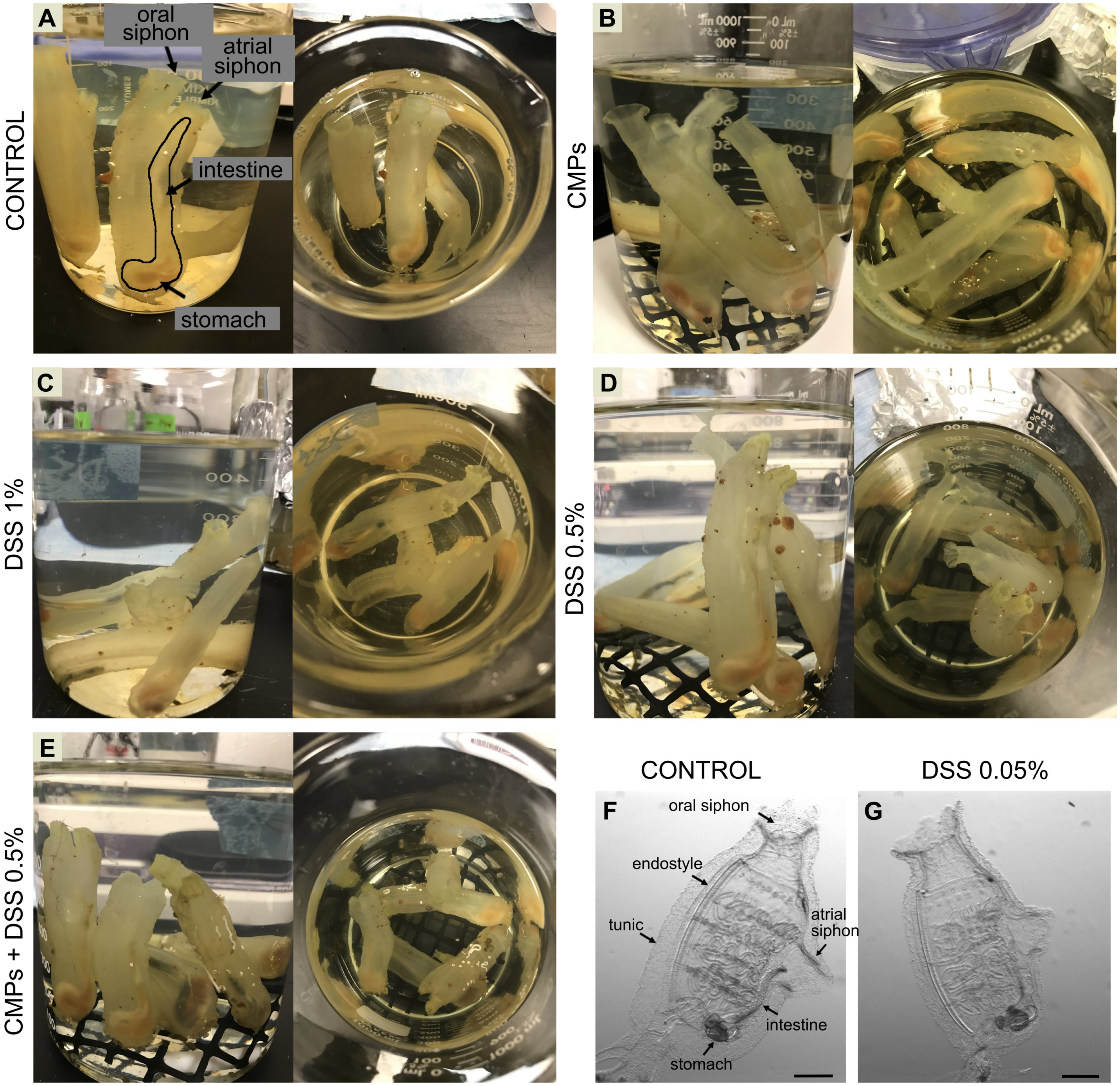

Figure S1. Ciona juveniles and adults. Whole mount images of *Ciona* adults after overnight treatment with the different experimental conditions. Control (A), CMPs (B), DSS 1% (C), DSS 0.5% (D), DSS 0.5% plus CMPs (E). Left image, side view; right image above view. Stage 7/8 of 1st ascidian juvenile control (F) and DSS 0.05% treated (G). n=30 animals observed for each condition. Scale bars 500 µm.

Biology Open • Supplementary information

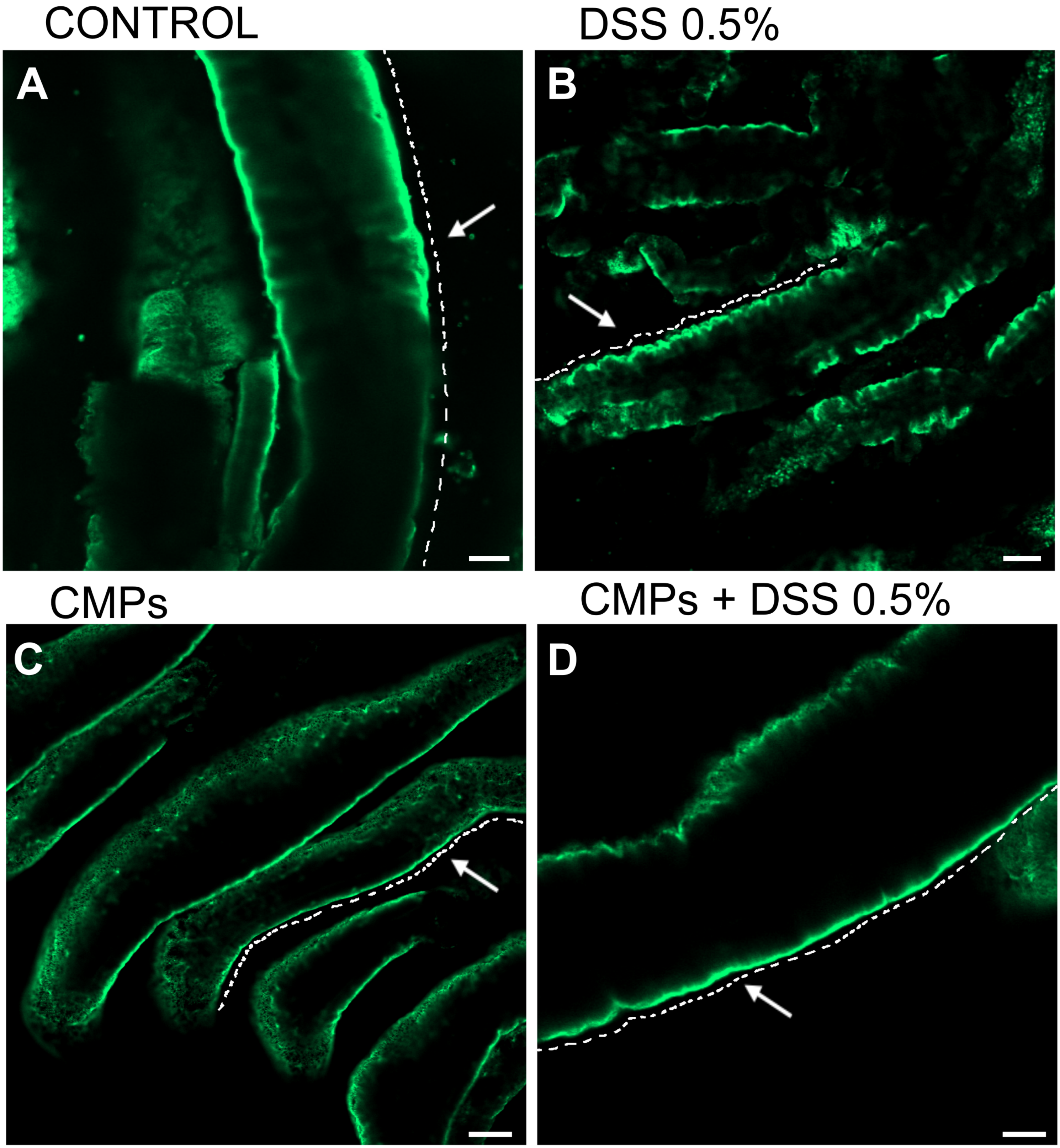
CONTROL
DSS 0.5%
A
B
CMPs
CMPs + DSS 0.5%
C
D

Figure S2. CMPs effect on the stomach of adult DSS-treated animals. Alexa Fluor 488 phalloidin staining reveals a smooth continuous stomach epithelium in control animals (A, arrow) and furrow-like structures in Control and (B) DSS-treated stomach epithelium (B, arrow). The presence of CMPs in the incubation medium did not affect the epithelial morphology (C D, arrow) and is comparable in to the controls, CMP-only treated animals (D C). White dashed lines highlight the morphology of the epithelium. n=6 animals observed for each condition. Scale bars (A, C D) 50μm and (B, D C) 100μm.
